# Diabetes-related changes in the protein composition and the biomechanical properties of human retinal vascular basement membranes

**DOI:** 10.1371/journal.pone.0189857

**Published:** 2017-12-28

**Authors:** Willi Halfter, Suzette Moes, Daphne O. Asgeirsson, Kathrin Halfter, Philipp Oertle, Esther Melo Herraiz, Marija Plodinec, Paul Jenoe, Paul Bernhard Henrich

**Affiliations:** 1 Department of Ophthalmology, University of Basel, Basel, Switzerland; 2 Proteomics Core Facility, Biocenter of the University of Basel, Basel, Switzerland; 3 Biocenter and the Swiss Nanoscience Institute, University of Basel, Basel, Switzerland; 4 Institute of Medical Informatics, Biometry and Epidemiology, Maximilian University Munich, Munich, Germany; Cedars-Sinai Medical Center, UNITED STATES

## Abstract

Basement membranes (BMs) are specialized sheets of extracellular matrix that outline epithelial cell layers, muscle fibers, blood vessels, and peripheral nerves. A well-documented histological hallmark of progressing diabetes is a major increase in vascular BM thickness. In order to investigate whether this structural change is accompanied by a change in the protein composition, we compared the proteomes of retinal vascular BMs from diabetic and non-diabetic donors by using LC-MS/MS. Data analysis showed that seventeen extracellular matrix (ECM)–associated proteins were more abundant in diabetic than non-diabetic vascular BMs. Four ECM proteins were more abundant in non-diabetic than in diabetic BMs. Most of the over-expressed proteins implicate a complement-mediated chronic inflammatory process in the diabetic retinal vasculature. We also found an up-regulation of norrin, a protein that is known to promote vascular proliferation, possibly contributing to the vascular remodeling during diabetes. Many of the over-expressed proteins were localized to microvascular aneurisms. Further, the overall stoichiometry of proteins was changed, such that the relative abundance of collagens in BMs from diabetic patients was higher than normal. Biomechanical measurements of vascular BM flat mounts using AFM showed that their outer surface was softer than normal.

## Introduction

Basement membranes (BMs) are thin sheets of extracellular matrix (ECM) that are located at the basal side of every epithelium. BMs outline muscle fibers and Schwann cells, and they are present at the basal surface of the vascular endothelial cells [1, 2]. BMs are comprised of approximately 30 high-molecular-weight glycoproteins that either polymerize or bind to other BM proteins to form thin ECM sheets [3, 4, 5]. Having emerged about 500 million years ago along with the evolution of metazoan species, BM components are the evolutionary oldest ECM proteins [6]. The biological and medical relevance of BMs is evident from phenotypes in worms, flies and humans bearing mutations of BM proteins that range from early embryonic death to muscular dystrophy, skin blistering, cardio-vascular defects, as well as severe eye, ear and brain abnormalities [7–10].

In 2003, diabetes affected approximately 200 million people worldwide [11]. While medication and recombinant insulin successfully treats hyperglycemia and ketoacidosis, the long-term chronic infections, such as diabetic nephropathy, neuropathy, retinopathy, and delayed wound healing, are not well controlled [12–14]. Diabetic retinopathy, for instance, affects 40% of all diabetic patients and is the leading cause of blindness under the age of 50 [15]. A histological) hallmark of long-term diabetes is thickening of BMs; it is particularly well documented for vascular BMs, but applies to other BMs as well [16–21].

Our laboratory has developed methods to isolate several human ocular BMs, including the retinal vascular BMs, and devised methods to investigate their cell adhesive and biomechanical properties, as well as an analysis of their protein composition [22–24]. In a recent project, we identified a series of diabetes-induced morphological, biophysical, and compositional changes by comparing retinal vascular BMs and inner limiting membranes from diabetic and non-diabetic human donors [21]. Expanding on earlier reports [18–20], we found that the thickness of ocular BMs increases by more than two-fold, both for type I and type II diabetes. The described changes in structural properties of diabetic BMs suggest that the protein composition of diabetic BMs is undergoing compositional changes as well.

The current study confirms the difference in protein composition of human retinal vascular BMs from diabetic and non-diabetic humans. Stiffness measurements using AFM showed that the outer surface of vascular BMs is softer—a fact that may account for the frequency of vascular aneurisms detected for the diabetic retinal vasculature.

## Experimental procedures

### Preparation of basement membranes

**Fig 1:**
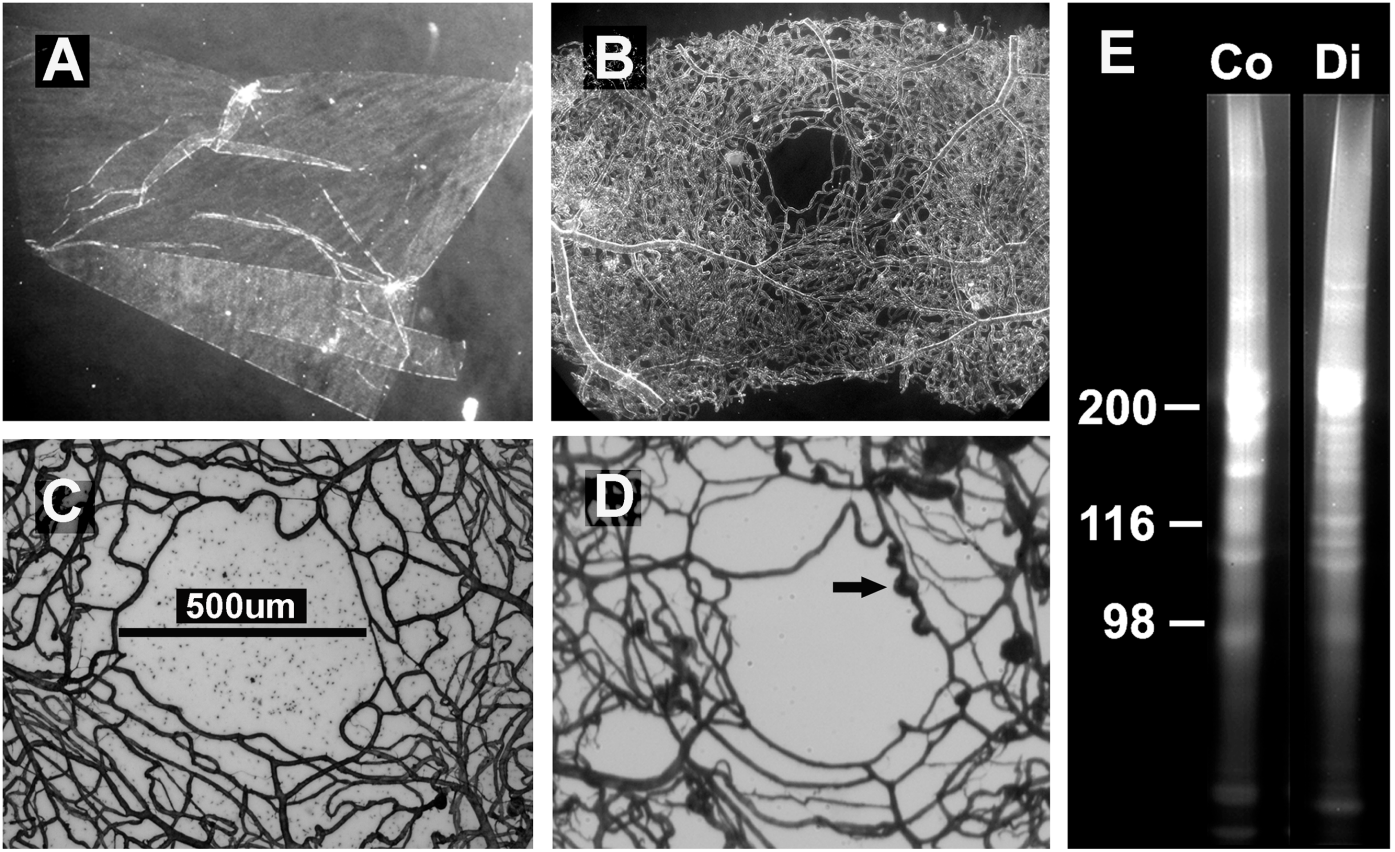
Isolation of BMs from human retina. Detergent-treatment of dissected retinas from human donor eyes resulted in the isolation of two in detergent-insoluble BMs from the retina. The ILMs appeared under the dissecting microscope and dark-field illumination as sheets that are often and at least partially curled up (A). The vascular BM sheets were distinguished from the ILMs by their flat, irregular and tubular appearance (B). Microscopic views of isolated vascular whole mounts, stained for collagen IV, are shown in (C) and (D). The whole mounts originated from the foveal area of each one non-diabetic (C) and one diabetic (D) eye. The diabetic vessels were more disorganized and presented numerous vascular aneurisms (arrow in D). Image (E) shows the SDS-PAGE analysis of vascular BM samples from a diabetic (Di) and a non-diabetic donor (n-Di), stained with Coomassie-Blue. Slightly different protein banding patterns indicated differences in the composition of the BMs.

Human donor eyes were obtained from CORE, the Center of Organ Recovery and Education (Pittsburgh, PA). The use of human eyes for this project was approved by the internal review board of the University of Pittsburgh under the IRB protocol number # 0312072. Written consent for use of the bodies and organs, including eyes, for transplantation and research was given by the participants of this study during their life time, or by their next of kin. The time intervals between death and organ harvesting ranged between 2 to 7 hours, their delivery to the laboratory taking place the next day after testing for HIV and hepatitis. None of the diabetic donor patients suffered from diabetic edema or proliferative diabetic retinopathy. The human retinal BMs were prepared as previously described [22–26]. In brief, retina were dissected from donor eyes, cut into squares of approximately 3x3 mm and incubated in a 10 cm dish without shaking in 2% Triton X100 at room temperature overnight. Solid Deoxycholate was added to a final concentration of 1%. After thirty minutes incubation, the detergent-insoluble BMs were repeatedly transferred into 10, 5 and 2.5 cm dishes with fresh Triton-X-100/deoxycholate solution by means of a Pasteur pipette under visual control with a dissecting microscope. The transfer into new detergent-solution removed retinal debris, organelles and cytoplasmic constituents, thus allowed detecting the free-floating, detergent-insoluble BMs more easily. After four transfers, the sheets of tubular blood vessel BMs were manually separated by means of a Pasteur-Pipette from the morphologically distinct sheets of the inner limiting membranes (ILMs) under a dissecting microscope using dark-field illumination (Fig 1A and 1B). Upon isolation, the BMs were stable and could be stored in PBS supplied with 0.01% sodium azide at 4°C. Since BM composition is known to change with age [22], BM samples from donors of similar age were analyzed.

### Digestion of basement membranes and proteomic analysis

Vascular BMs from both eye globes of one patient were spun at 10,000 rpm for 3 min, washed 3 times, and the pellet was taken up in 150 μL of PBS. 50 μL of collagenase (1000U/mL; type VII; Sigma) were added, and the sample incubated for 24 hours at 37°C. Next, the proteins were reduced with 10 mM DTT at 37°C for 1 hour and alkylated with 50 mM iodoacetamide for 15 min at room temperature. Protein digestion was performed by incubation of the sample with 1 μL of trypsin (Sequencing grade, Promega) at 37°C overnight. This digestion regime resulted in a complete solubilization of the BMs. The digest was desalted on a microspin column (The Nest Group, Southborough, MA, USA) according to the manufacturer’s recommendations. Peptide absorbance was measured at 280 nm and peptide concentration was calculated according to [27]. LC/MS/MS analysis was performed on either an Orbitrap Elite or Orbitrap Classic (Thermo, Scientific, Reinach, Switzerland) interfaced with an EASY-nLC 1000 pump connected to a C18 column (75 μm x 15 cm) packed with 2.4 μm Reprosil particles [26]. For each analysis, equal peptide material (2 μg) was injected in triplicates onto the capillary column. Chromatography and mass spectrometric parameters corresponded to methods described in [28].

The LC/MS/MS data were searched against the human databank from SwissProt. The databank was updated monthly. The Mascot and Sequest HT search engines were run via Proteome Discoverer 1.4 (Thermo Scientific). Search parameters were set to carbamidomethylated cysteines as fixed modification, while oxidized methionines, and protein N-terminal acetylation were selected as variable modifications. Peptides were accepted that had a false discovery rate below or equal than 1%. For relative protein quantification, the area under the curve for identified peptides for a given protein was calculated using Proteome Discoverer 1.4. For differences in the two proteomes, we compared the BM proteomes of 3 pairs of diabetic eyes and 3 pairs of age-matched non-diabetic donor eyes. Each data set was analyzed in three technical replicates. In each of the three LC-MS/MS runs, we analyzed one sample from a diabetic and one from a non-diabetic donor (see brackets in the first column of Tables 1 and 2).

**Table 1:** Paired samples used for the MS analysis of vascular BMs.

Age/gender	Cause of death	Diabetes Duration	μg peptide	# of proteins	# of ECM proteins	% ECM
**54y m (1)**	**MI**	**-**	**47**	**89**	**32**	**36%**
***Di 52y m (1)***	***Peritonits***	***IDDMx17y Type II***	***35***	***109***	***46***	***42%***
**64y m (2)**	**AAA+MI**	**-**	**25**	**110**	**47**	**43%**
***Di 54y m (2)***	***CVA***	***IDDMx30y Type II***	***40***	***204***	***79***	***39%***
**47y m (3)**	**SIGSW**	**-**	**15.4**	**45**	**28**	**62%**
***Di 38y m (3)***	***CVA***	***IDDMx30y Type I***	***15*.*4***	***95***	***47***	***49%***

The table lists age of the donors, their gender, cause of death, duration of diabetes affection (x years of diabetes), the peptide yield for the vascular BM samples after collagenase and trypsin digestion (in μg), the total number of proteins, the number of ECM proteins and the percentage of ECM proteins relative to the total number of proteins. SIGWSL: self-inflicted gunshot wound; MI: myocardial infarction; AAA: abdominal aortic aneurism; CVA: cerebral vascular accident. The numbers in the first column (brackets) indicate which pairs of BM samples were analyzed together.

**Table 2:** Protein classes as detected in non-diabetic and diabetic (Di) vascular BM samples.

age	total proteins	ECM	cytoskeletal	nuclear	cytoplasmic
**54 (1)**	**89 = 100%**	**32 = 49%**	**9 = 27%**	**3 = 4.5%**	**3 = 5.6%**
**Di52 (1)**	**109 = 100%**	**46 = 68%**	**14 = 9.9%**	**7 = 4.0%**	**15 = 9.8%**
**64 (2)**	**110 = 100%**	**47 = 76%**	**11 = 8.6%**	**3 = 4.1%**	**9 = 7.5%**
***Di54 (2)***	***204 = 100%***	***79 = 61%***	***7 = 10*.*3%***	***6 = 10*,*1%***	***14 = 9*.*8%***
**47y (3)**	**45 = 100%**	**28 = 77%**	**6 = 9.7%**	**nd**	**4 = 7.7%**
***Di38 (3)***	***95 = 100%***	***47 = 73%***	***11 = 5*.*5%***	***2 = 0*.*6%***	***13 = 9*.*3%***

Row one identified the donors according to their age. The BM samples are organized as analyzed in pairs from each a non-diabetic and a diabetic donor (shown in brackets). Di: diabetic donor (grey) as listed in Table 1. Row two lists the total number of proteins that account for 100% of the total protein. Rows three to six lists the number of ECM, cytoskeletal, nuclear and cytoplasmic proteins and their percentage relative to the total protein content of the samples.

### Immunocytochemistry

For immunocytochemistry, sheets of vascular BMs were spread onto Superfrost-plus slides (Fisher Scientific, Waltham MA) coated with 5μg/mL poly-lysine (Sigma, St. Louis, MO). For firm attachment of the BM sheets, the slides were centrifuged at 1000 rpm for 5 minutes. The whole mounts were washed twice with 1% BSA, 0.01% Triton-X-100 and stained with polyclonal antisera to collagen IV (Rockland, Gilbertsville, PA), laminin (Sigma, St. Louis, Mo), agrin (kindly provided by Dr. Gregory J. Cole [29]), or a mouse monoclonal antibody to the 7S-domain of collagen IV α3 (Mab J3-2; kindly provided by Dr. Nirmala SundarRaj [30]). The specified IgM antibody is also available from Sigma Aldrich (SAB4200500). Polyclonal, affinity-purified rabbit antibodies to norrin (NBP1-59305), C9 (NBP2-15952) and ApoE (NBP1-89033) were purchased from Novus (Novus, Biologicals Europe, Abingdon, UK). A monoclonal antibody to fibronectin (Mab1949) was obtained from Millipore (Temecula CA). The polyclonal antiserum to PRELP was a generous gift from Peter Rugley and Jody Summers [31]. Secondary antibodies were Cy3 or Alexa-Fluor 488-labeled goat ant-rabbit, goat anti-mouse, or mu-chain-specific goat anti-mouse antibodies (Jackson ImmunoResearch, West Grove, PA; and Life Technologies, Carlsbad CA). Micrographs were taken with an Olympus Flow-view confocal microscope. Each of the antibodies was tested with three BM samples from diabetic and with three BM samples from non-diabetic donors. Samples used for immunocytochemistry were taken from the same human donors as the samples used for the proteomic analysis, listed in Table 1. Samples from diabetic and non-diabetic donors were always processed and recorded on a microscope simultaneously. The sections were first pre-viewed with a conventional compound fluorescent microscope for positive or negative staining. For an unbiased documentation between diabetic and non-diabetic samples with the confocal microscope, we photographed the BM samples with a clear positive staining first, and then used the identical microscope setting for the microscopic documentation of the corresponding control sample. Each staining procedure and documentation session was repeated at least twice to confirm reproducibility.

### Atomic Force Microscopy (AFM)

For stiffness measurements, sheets of vascular BMS were spread onto Superfrost-plus glass slides (Fisher Scientific, US) or 2 cm tissue cultures dishes (TPP AG, Switzerland) and firmly mounted onto the substrates by centrifugation as described for immunocytochemistry. The measurements were performed using a custom built mechano-optical microscope (MOM) based on a JPK Nanowizard I (JPK AG, Berlin, Germany) operated with a Nanonis controller (Specs Zurich, Zürich, Switzerland) mounted on a Leica DMI8 (Leica Microsystems, Heerbrugg, Switzerland). Prior to force spectroscopy measurements, the cantilevers spring constant was determined using the Sader-method [32] and the deflection sensitivity was calibrated on a culture dish. Force Spectroscopy was performed with a load of 3.1 nN at an indentation speed of 16 μm/s. Force- curves were analyzed with the custom written ARTIDIS READER, using the Oliver—Pharr model [33] to calculate the elastic modulus. Significance was tested using the STUDENT’S t-test.

## Results

### 1. BM isolation from human eyes

For proteome analysis, we isolated the retinal vascular BMs from three pairs of diabetic and three pairs of non-diabetic cadaver donor eyes (biological repeats, n = 3; each). BM isolation involved retina dissection followed by treatment of the retinal squares with detergent [22–26]. While the detergent treatment of the retinal segments resulted in the solubilization of all cellular components of the retina, the detergent-insoluble inner limiting membranes (ILMs) and vascular BMs remained intact as free-floating ghosts in the detergent solution. A prior purification of the retinal vasculatures as described by Kuwabara and Cogan [34] was not required for the isolation of the retinal vascular BMs, but the ILMs dad to be manually separated from the vascular BMs under a dissecting microscope. The ILM appeared under dark-field illumination as translucent sheets that were often curled up (Fig 1A). They were morphologically distinct from the multi-tubular vascular BM sheets (Fig 1B). Transmission and scanning electron microscopy imaging [22–24] showed that the isolated vascular BM sheets were clean ECM structures and not contaminated with nuclear or cellular debris. Additional evidence for the absence of cellular component came from vascular whole mounts stained with CytoxGreen, a generic nuclear dye that showed no intact or fragmented nuclei (not shown). Likewise, proteomics data confirmed the very low abundance of nuclear proteins in the proteome data lists (S1–S6 Tables; each sheets A). Segments of the intact retina, from which the BMs were isolated, had previously been analyzed for diabetes-typical morphological changes [21]. Age, gender, and medical history, including cause of death of the donors have been listed as well [21]. Consistent with earlier publications, (16–21), the diabetic and non-diabetic vascular BMs used in this study were thicker than normal [21] and showed characteristic microvascular aneurisms (Fig 1D) that were not detected in vascular BM whole mounts from non-diabetic eyes of this age (Fig 1C). Further, the overall arrangement of the capillary BM tubes from diabetic donors appeared more disorganized than in non-diabetic eyes (compare Fig 1C and 1D). SDS-PAGE analysis showed slightly different protein banding patterns for diabetic and non-diabetic BM proteins (Fig 1E). For an unbiased and comprehensive identification of the protein differences, vascular BM samples from diabetic and non-diabetic eyes were analyzed by LC-MS/MS and the identified protein were compared.

### 2. Analysis of human retinal vascular BMs from non-diabetic donors

For a simplified LC/MS/MS analysis of human retinal vascular BMs, we used a novel sample preparation technique. For this, solid BM samples were first digested with collagenase, followed by a second round of trypsin digestion. The combined enzyme treatments led to a complete solubilization of the BMs verified by the absence of any insoluble material after centrifugation and microscopic inspection. Due to this unprecedented method, we first analyzed the protein composition of vascular retinal BMs from non-diabetic donors to calibrate a baseline for the comparison to the proteome of BMs from diabetic donors. The analysis of non-diabetic BMs and its comparison with data from a previous, more elaborate sample preparation approach also established the reliability of the new preparation. Results showed that the peptide yield from the three pairs of non-diabetic eyes ranged between 15 and 47μg total peptide (mean 36±15, n = 3; Table 1). Six micrograms were sufficient for the three technical LC/MS/MS replicate runs. Table 1 also lists age, gender and the cause of death of the donors.

Using a stringent 1% peptide false discovery rate cutoff, we identified between 45 and 110 proteins for these samples (mean 99±15, n = 3; S1–S3 Tables; each sheets A). Between 28 and 47 of the identified proteins were ECM constituents (mean: 36±10), accounting for 36 to 62% of the identified proteins (mean: 40±5%; Table 2 and S1–S3 Tables; each sheets B).

The relative concentrations of the individual proteins in each of the non-diabetic BM samples were calculated by measuring the area under the curves of the identified peptides that were detected for each protein. The abundance of a given protein was expressed as percentage relative to the total protein that was set to 100% for each sample. The 28 to 47 ECM proteins accounted between 49% and 77% of the total encountered protein (mean: 67±15%, Table 2, grey lines). The main non-ECM proteins were cytoskeletal constituents (15±10%), followed by cytoplasmic (2.8±2.4%) and nuclear proteins (6.9±1.1%; Table 2).

**Fig 2:**
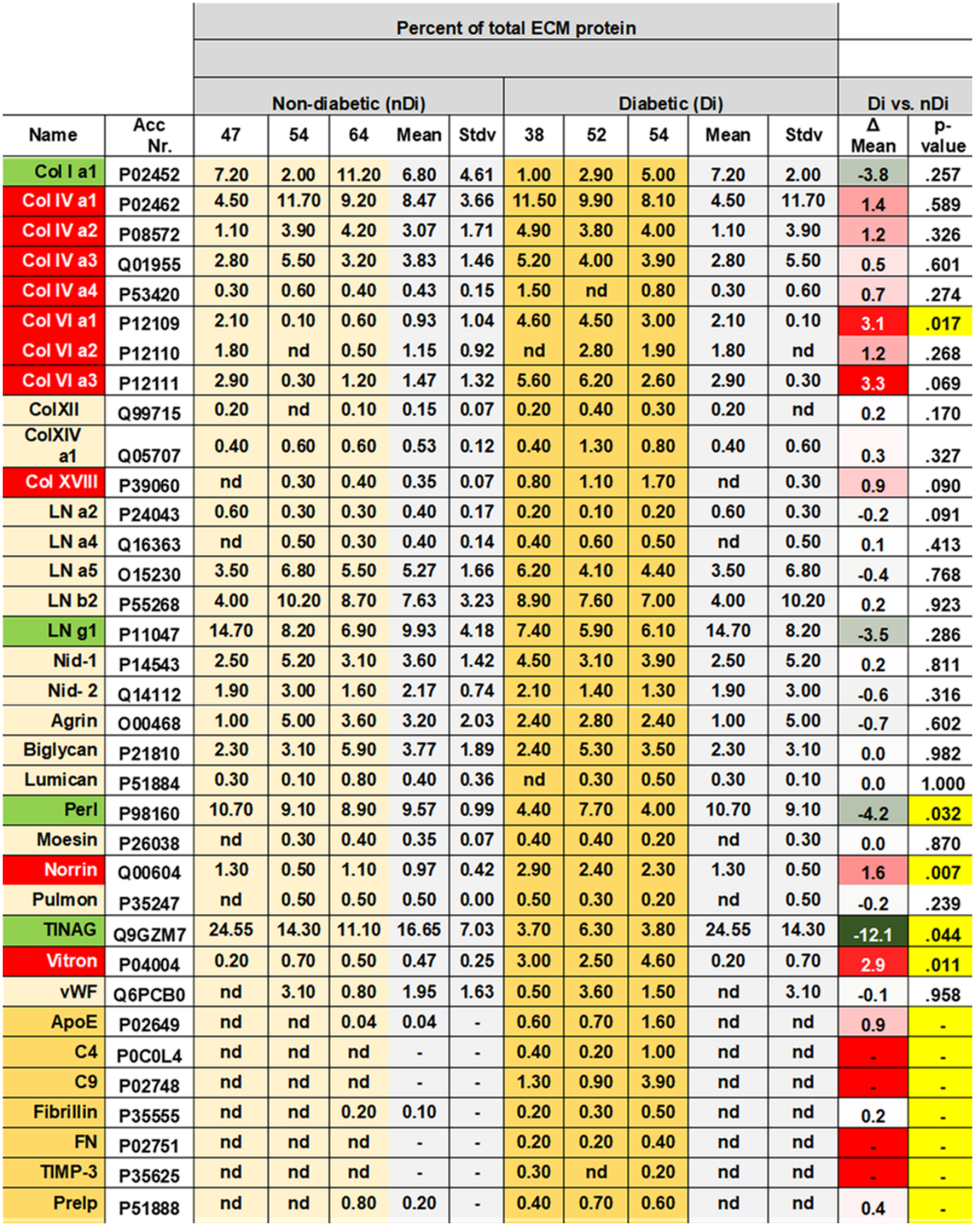
MS-identified proteins from vascular BMs of three diabetic and three non-diabetic donors as listed in Table 1. The abundance of a protein was determined by calculating the area under all peptide peaks from the identified protein and expressed in % of total ECM proteins. Proteins labeled in green have a lower relative abundance in diabetic as compared to non-diabetic patients while candidates marked in red exhibit a higher abundance. Individual values are listed for all three non-diabetic and all three diabetic samples. The means of all three samples was calculated with the standard deviation, and the difference between diabetic and non-diabetic values are listed as well. The p-values for these differences are also listed. The p-values in yellow are statistically significant. The proteins listed in yellow were considered typical to diabetic BMs. Di. Diabetic. nDi. Non-diabetic, Stdv, Standard deviation. Perl: Perlecan, Vtron: Vitronectin; Nid 1 and Nid 2: Nidogen 1 and 2. Pulmon: Pulmonary surfactant protein; vWF: von Willebrand Factor.

Non-ECM proteins were subtracted from the proteome data of each sample to obtain a list of only ECM proteins (S1–S3 Tables; each sheets B). To quantify the individual ECM proteins for each sample, the entire ECM proteome was set to 100%. The abundance of the identified individual ECM protein was calculated as percentage relative to the 100% of the ECM proteome per sample (S1–S3 Tables; sheets A and B and pie-charts on S1–S3 Tables, sheets B). The lists of identified ECM proteins of all three samples were compared, and proteins encountered in all three samples were then selected as reliable BM proteins of non-diabetic adult human retinal vascular BMs. Twenty-eight ECM shared ECM proteins were identified in each of the analyzed vascular BM samples. The human vascular BM proteome included four collagen IV (12.7% of the total ECM proteome) and three collagen VI peptide chains (3.6%), five laminin peptide chains (24.6%), nidogen I (3.6%) and II (2.2%), and the proteoglycans perlecan (9.6%), agrin (3.2%), collagen XVIII (0.4%), biglycan (3.8%) and lumican (0.4%). A major component of the vascular BMs was tubulointerstitial nephritis antigen, TINAG, a BM protein that had previously been identified in kidney tubular BMs (16.7%), [35, 36]. Additional protein components were collagen Iα1 (6.8%), collagen XII (0.2%) and XIV (0.5%), moesin (0.4%), norrin (1%), pulmonary surfactant protein (0.5%), virtronectin (0.5%), and von Willebrand factor (2%; see Fig 2).

### 3. Comparison of human retinal vascular BM from diabetic and non-diabetic donors

The peptide yield from the solubilized human retinal vascular BMs of diabetic donors was between 15 and 40 μg (mean 30±13 μg). Between 95 and 204 proteins were identified (mean 136±59; S4–S6 Tables; each sheets A), and between 79 and 46 of those were ECM proteins (mean 57±19; S4–S6 Tables; each sheets B). The ECM proteins equaled between 39% and 49% of the total proteins identified (mean 43±5%). The relative quantity of each of these ECM proteins is listed in S4–S6 Tables and the pie-charts on sheets B of S4–S6 Tables. To compare the proteomes from non-diabetic and diabetic human donors, the relative quantities of ECM proteins verified in all three diabetic BM samples were selected as described above. The identified proteins and relative quantity data sets from the diabetic and non-diabetic samples were then directly contrasted as shown in Fig 2. The Figure also includes 5 proteins that were only found in two of the three non-diabetic samples and in three of the diabetic samples, and three that were only found in two of the diabetic samples and in three of the non-diabetic samples. Fig 2 further shows that all of the twenty-eight proteins that were identified in the non-diabetic BM samples were also detected in all BM samples from diabetic donor eyes. The shared human vascular BM proteome from diabetic donors included four collagen IV (19.6% of the total ECM proteome) and three collagen VI peptide chains (11.2%), five laminin peptide chains (19.9%), nidogen I (3.8%) and II (1.6%), and the proteoglycans perlecan (5.4%), agrin (2.5%), collagen XVIII (0.4%), biglycan (3.7%) and lumican (0.4%). TINAG tubulointerstitial nephritis antigen, was present with 4.6%, collagen Iα1 with 3%, collagen XII with 0.2%, collagen XIV with 0.5%, moesin with 0.3%, norrin with 2.5%, pulmonary surfactant protein with 0.3%, virtronectin with 3.4%, and von Willebrand factor with 1.9% (Fig 2).

We found seven proteins to be present in diabetic BMs samples, but were undetectable in equivalent specimen from non-diabetic BM samples. These proteins we considered to be diabetes-typical, including two members of the complement system, C4 and C9, ApoE, fibrillin, fibronectin, TIMP-3 and Prolargin/PRELP, a proteoglycan. Ten proteins were found to be more abundant in diabetic than in non-diabetic BMs, including several collagen IV and VI family members, collagen XVIII, norrin and vitronectin. Four proteins were more abundant in non-diabetic than diabetic BMs: collagen Iα1, laminin γ1, perlecan, and TINAG.

**Fig 3:**
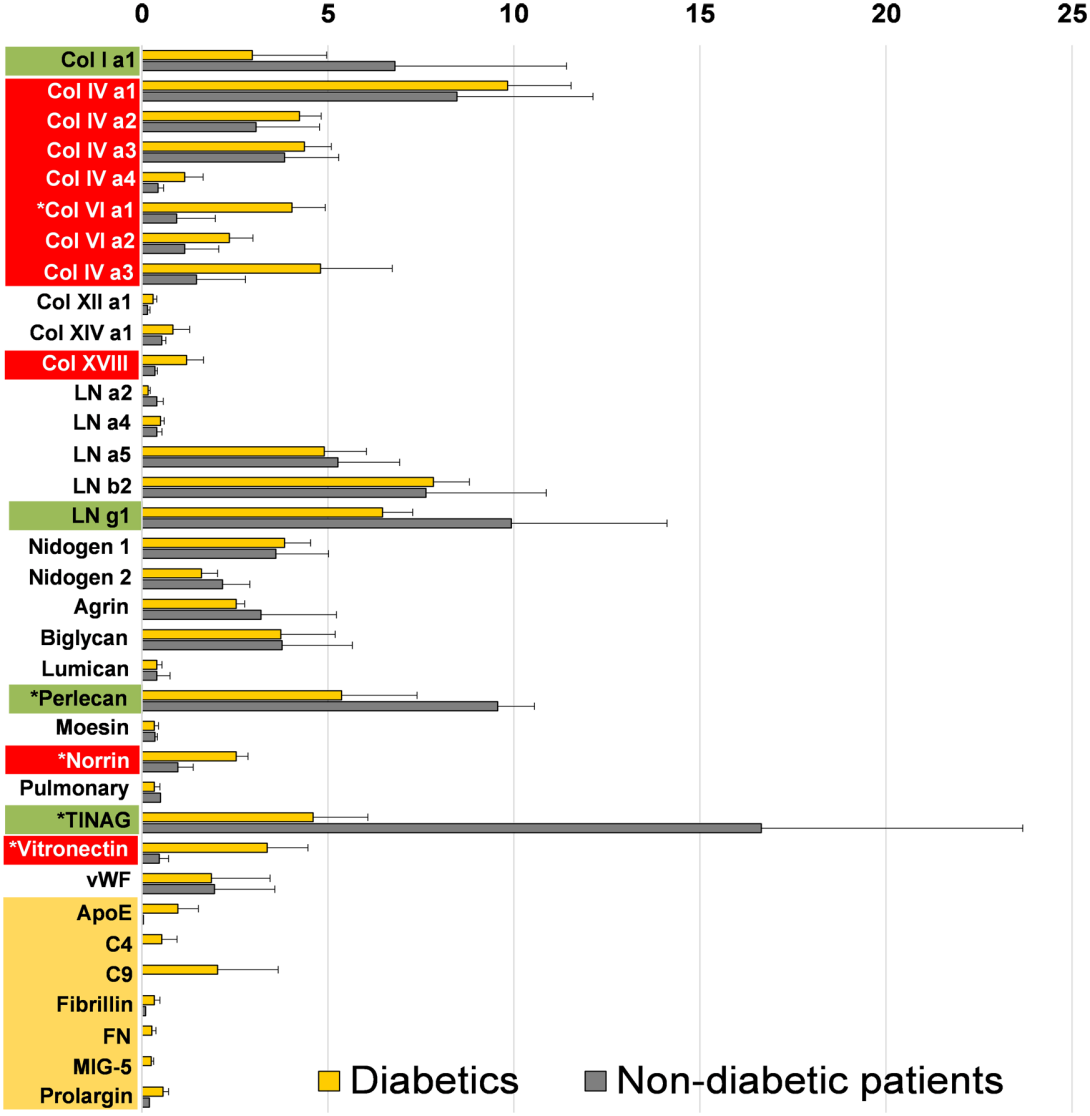
Graphic representation showing the relative abundance of BM proteins in vascular BMs from diabetic (yellow bars) and non-diabetic donors (grey bars) in percent, relative to the entire ECM proteome set to 100%. The means from three diabetic and three non-diabetic BM analyses with standard deviation are shown. The proteins that are more abundant in diabetic as compared to non-diabetic BMs are labeled in red, the proteins less abundant in green. White stars mark statistically significant differences. The proteins in yellow at the bottom of the graph were considered diabetes-typical BM proteins.

Fig 3 graphically summarizes the described data. The bar graph compares the mean abundances of proteins present across all diabetic and all three non-diabetic donor samples. Data infer that the most prominent laminin family member in vascular retinal BMs is LNα5β2γ1. The most abundant collagen IV member is ColIVα1α1α2, while perlecan is the most abundant proteoglycan, followed by biglycan, agrin, collagen XVIII and lumican.

As shown in Fig 3, members of the collagen families IV, VI and XVIII have a greater abundance in diabetic BMs, yet the difference was statistically significant for Col VIα1 only. Such statistically significant differences were also observed for vitronectin and norrin. Diabetes-typical proteins were listed separately at the bottom of the graph, and the differences between diabetic and non-diabetic BMs were statically significant for all seven compounds. From the five proteins that are detected in non-diabetic and diabetic BMs, but were more abundant in non-diabetic than in diabetic BMs, the differences for perlecan and TINAG were statistically significant.

We also calculated the abundance of collagen family members, laminins and all proteoglycans in vascular BMs of diabetic and non-diabetic donors, thereby defining the total protein content of each sample as 100%. Results showed that the abundances of all collagens, including collagen IVs and VI were elevated in diabetic BMs, while the abundance of all laminin members was reduced (Table 3). By calculating the ratios of total collagens vs. total laminins or nidogen I, we found that the overall BM protein stoichiometry changed during diabetes to a higher relative concentration of collagens (Table 3). The relative abundances of laminins and proteoglycans were reduced. The ratio of the total collagen content in general, and collagen IV in particular, to laminin was increased by more than 60% in diabetic BMs. Further, the relative abundance of proteoglycans relative to nidogen 1 decreased by at least 60%.

**Table 3:** Table listing the relative abundances (grey; in percent relative to the total protein) of all collagens, collagen IVs, VIs, and α1, LNs, proteoglycans.

% of total protein or ratio	Non-diabetic	Diabetic
***All collagen family members***	***27%***	***36%***
***Collagen IVs*** ***Collagen Vis*** ***Collagen α1***	***16%*** ***3*.*6%*** ***6*.*8%***	***21%*** ***11*.*2%*** ***3%***
***All LN family members***	***24%***	***20%***
***All Proteoglycans***	***17%***	***12%***
**Ratio Collagens/LNs**	**1.1**	**1.8 = 60% increase**
**Ration Collagen IV/LN**	**0,7**	**1.0 = 67% increase**
**Ration Collagens/Nidogen I**	**7.2**	**9.5 = 8% increase**
**Ration Collagen IVs/Nidogen I**	**4.3**	**5.4 = 8% increase**
**Ratio Proteoglycans/Nidogen 1**	**4.7**	**3.1 = 66% drop**

The ratios (yellow) of collagens vs. LN, collagen IVs vs LN, collagens vs nidogen I, collagen IVs vs. nidogen I and proteoglycans vs nidogen I in diabetic and non-diabetic vascular BMs are listed as well.

### 4. Distribution of diabetes-specific ECM proteins

We used immunocytochemistry to validate the up-regulation of proteins in diabetic versus non-diabetic vascular BMs as found in the proteome analysis. Special attention was hereby paid to the microvascular aneurisms, being a hallmark for diabetic vascular BMs (Fig 1). The whole mounts of vascular BMs also allowed for the identification of sub-regions in the vascular system, such as sprouts or aneurisms that would have been easily missed by using tissue sections. Polyclonal antisera to laminin, collagen IV and agrin stained uniformly all vascular BMs, including microvascular aneurisms, and they were abundantly present in both diabetic and non-diabetic BMs (not shown).

**Fig 4:**
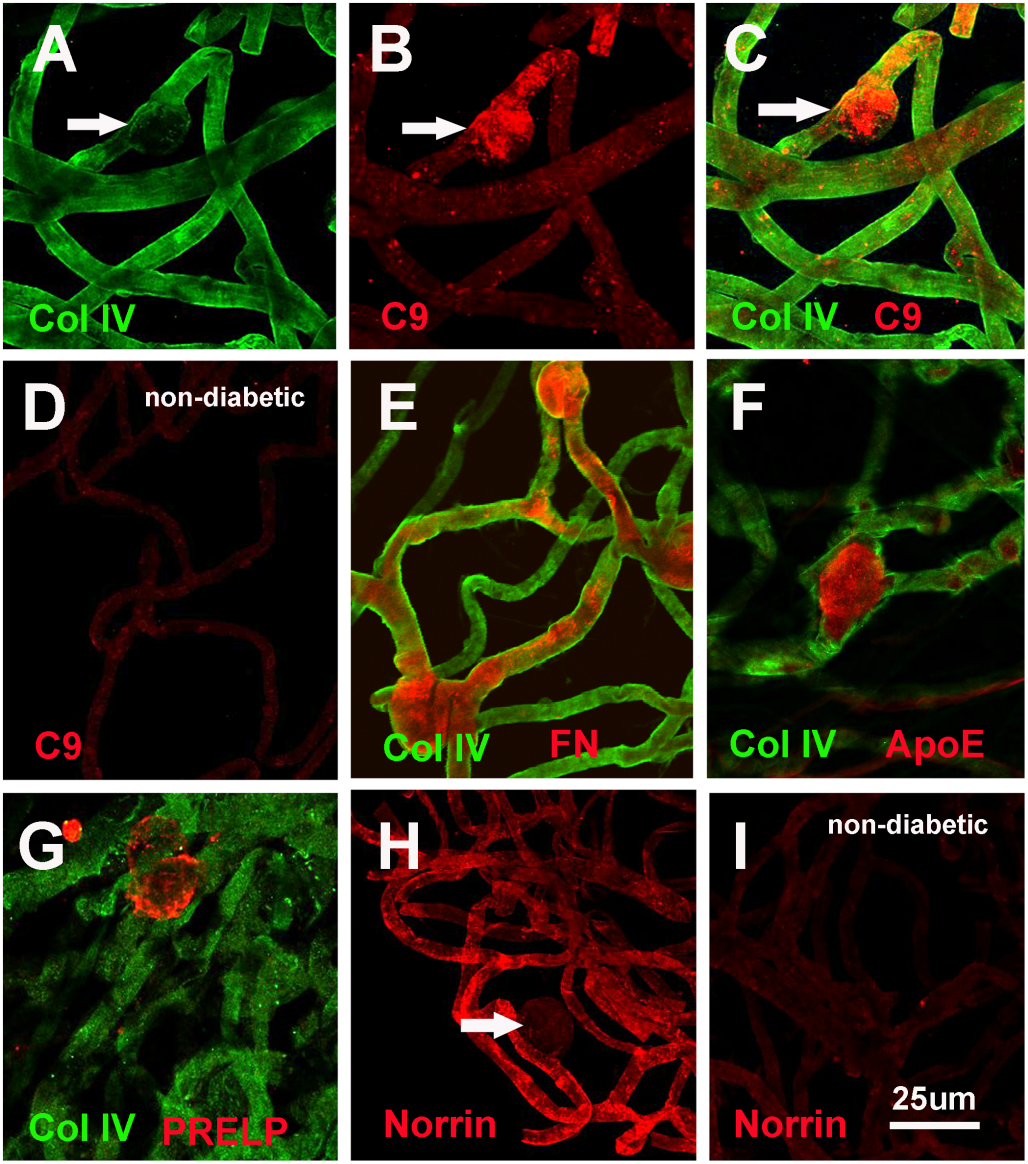
Staining of vascular BM whole mounts with antibodies to proteins detected in the proteome analysis. A generic staining of the vascular BMs was given by an antibody to the 7S domain of collagen IV α3 (A, C, E, F, G). Prominent staining for microvascular aneurisms was detected by staining with antibodies to C9 (B, C), Fibronectin (FN, E), ApoE (F) and PRELP (G). The same treatment of vascular BM whole mounts from non-diabetic eyes did not show staining for these proteins (D). A norrin-specific staining is shown to be generic for the entire vascular BM whole mounts (H), the signal, however, being less prominent in vascular aneurisms (arrow in H). Staining of vascular BM whole mounts from non-diabetic eyes showed a clearly weaker staining for norrin, when compared to vascular whole mounts from non-diabetic donors. Bar: 25um.

A monoclonal antibody to the 7S domain of collagen IV α3 labeled the vascular BMs from both diabetic and non-diabetic eyes; staining of the microvascular aneurisms, however, was by far weaker in diabetic BMs than compared to the non-diabetic control samples (Fig 4A). This antibody was therefore used as a generic stain to outline of the course of the vascular BM tubes. Several antibodies to proteins that are upregulated according to the proteome analysis prominently stained the microvascular aneurisms, as exemplified by staining with an antibody to complement factor C9 (Fig 4B and 4C). Vascular whole mounts from non-diabetic eyes showed very little C9 staining (Fig 4D). Specific staining for aneurisms in diabetic BMs whole mounts was also seen with antibodies to fibronectin (4E), ApoE (Fig 4F) and PRELP (Fig 4G). In contrast to this, anti-norrin antibodies lead to an overall brighter signal for diabetic (Fig 4H) versus non-diabetic vascular BMs (Fig 4I). For norrin, no staining was detected in vascular aneurisms (Fig 4H, arrow).

### 5. Biomechanical testing of vascular BMs using Atomic Force Microscopy

**Fig 5:**
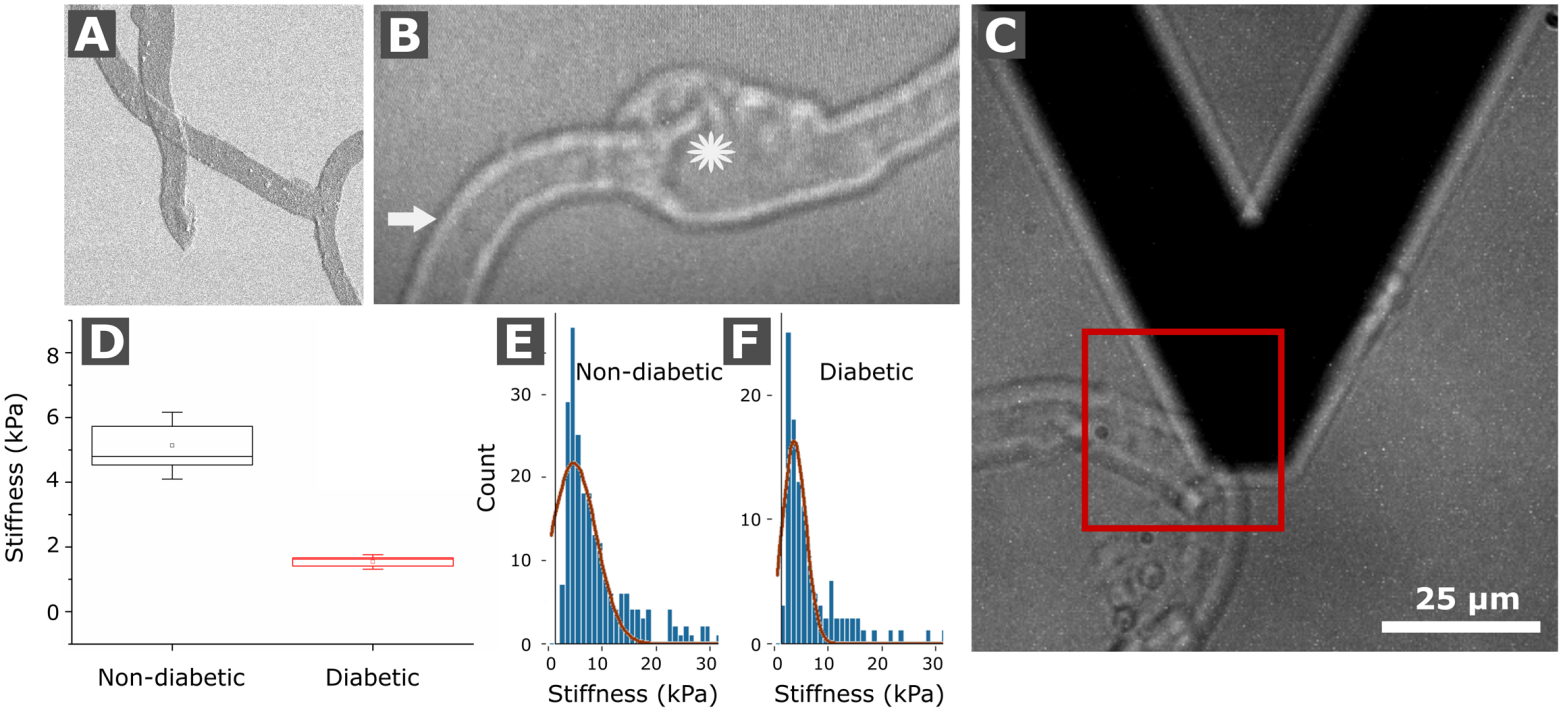
Biomechanical testing of human retinal vascular BM whole mounts by AFM. **(A)** Image shows a SEM micrograph of flat-mounted vascular BM sheets. (B) A representative light micrograph from a flat-mounted capillary during AFM measurements is shown. The star hereby marks an aneurism while the arrow indicates the region of AFM data collection on the sample surface in order to probe the biomechanical stiffness of a capillary. (C) Panel shows the AFM probe next to a capillary that was about to be probed in the indicated box. The box plot in (D) contrasts the average stiffness of non-diabetic and diabetic vascular BMs, respectively. Representative graphs show the relative frequency of AFM stiffness values for non-diabetic vascular BMs (E) and diabetic vascular BMs (F).

To investigate whether changes in the protein composition of the vascular BMs from human retinal blood vessels are accompanied by alterations of their biomechanical properties, retinal vascular sheets were whole-mounted and subsequently probed for stiffness using Atomic Force Microscopy (AFM). Fig 5A shows a Scanning Electron Microscope (SEM) view of the mounted sheets to demonstrate that the preparation is not visibly contaminated by non-ECM tissue and cellular debris. A light microscopy view of a capillary is shown in panel (Fig 5B). Only well separated capillaries were tested by AFM (5C). Further, it is of note that only the stiffness of the outer surface of the capillary BMs and not the inner surface was accessed in this particular assay. Due to the low number of tested aneurisms, stiffness data from microvascular aneurisms was not included in this project.

We mechanically probed samples from three non-diabetic human retinas and three analogous specimens from diabetic human donors. Stiffness data was generated and confirmed in three different data collections. The mean stiffness of the vascular BMs from non-diabetic donors was 5.1± 1.03 kPa (n = 3 different samples; Table 4 and Fig 5D), the mean stiffness from diabetic donors was 1.53±0.23 kPa (n = = 3 different samples; Table 4 and Fig 5D). With a factor of 3.4, the difference in AFM-measured stiffness between diabetic and non-diabetic BM stiffness, respectively, was statistically significant (p<0.05; Fig 5D).

**Table 4:** Shows the AFM based stiffness data from three different vascular BM flat mounts (numbered 1 to 3).

	Non-diabetic	Diabetic
1	4.30±1.7 kPa	1.62±0.8 kPa
2	6.29±1.6 kPa	1.28±0.6 kPa
3	4.80±2.7 kPa	1.70±0.8 kPa

From each sample, at least three different capillaries were assayed. Data is given as means with standard deviations.

## Discussion

### 1. Technical considerations

We compared the proteomes of retinal vascular BMs from adult human diabetic and non-diabetic donors. Since the composition of human retinal BMs changes with advancing age [22], vascular BM samples of middle-aged patients, ranging from 38 to 64 years of age, were used for this comparison. Previously, BM proteins had been solubilized for LC-MS by boiling the samples in high molar urea and SDS, and the solubilized proteins were separated by SDS-PAGE. The individual protein bands were then excised prior to individual analysis. This procedure was time-consuming and required extended LC/MS time [5, 37]. In this study, we introduce a novel method for sample preparation, where the vascular BM tubes are digested with bacterial collagenase and subsequently exposed to trypsin. This combination of enzyme treatments led to a complete solubilization of the BMs and allowed to directly analyze the released peptides by one-shot LC-MS/MS analysis runs. Take notice that either of the enzyme treatment alone was insufficient for BM solubilization. Albeit the set of proteins identified by using the described sample digestion procedure was shorter than the list we obtained from the more laborious SDS-PAGE technique (5). Nonetheless, all the major ECM proteins identified in the abbreviated sample preparation nearly greatly corresponded to the proteins identified before, and none of the major BM components being missed. The mean percentage of ECM proteins relative to non-ECM protein contaminants was even higher (45% versus 16% [5]). The same was true for the abundance of ECM proteins relative to total protein: the ECM proteins identified with the current analysis accounted for 67% of total protein, whereas in the previous analysis the ECM proteins accounted for only 46% of the total protein [5]. With the current technique, we even identified a protein that had been missed in our previous, more laborious study: TINAG, a BM protein known from the analysis of kidney BMs [35, 36]. In fact, TINAG turned out to be one of the most prominent proteins in diabetic and non-diabetic vascular BMs. Consistent with earlier results, the vascular BMs included several collagen IV and laminin family members, respectively. The most prominent of these were the collagen IVs, of which particularly the alpha chain combination α1α1α2 was the most prominent representative [38]. The second most prominent protein was TINAG. The third most prominent members were the laminins with the main chain composition of α5β2γ1, followed by nidogen 1 and 2, and several proteoglycans, with perlecan as the dominant family member [39]. A problem in the earlier analysis was that the quantity data did not always reflect the expected chain compositions of collagens and laminins [5, 37]. In the current study, we found that the relative amount of collagen IVα1 was twice that of collagen IVα2, the expected ratio. Likewise, the ratio of collagen VI and laminin α5, β2 and γ1 was in similar range. However, there were also unexpected data: for example, we found collagen IV α3 and α4 but no α5. Also, we found a substantial amount of collagen Iα1 but very little of the expected collagen Iα2. It is likely that not all proteins fragment as efficiently and went, for this reason, undetected or were under-represented. However, preliminary experiments examining the proteomes of the inner limiting membrane, the lens capsule and Descemet`s membrane from human eyes showed that collagen IV α5 is readily detectable in similar quantities as the α3 and α4 chains [Suzette Moes, Willi Halfter, in preparation]. It is also conceivable that the collagens may assemble into trimers that have previously not been considered, for example to a collagen IVα3α3α4 hetero-trimer, or even an IVα3α3α3 homo-trimer. Another caveat with the current sample preparation is that the abundance of collagens might be underestimated, since collagenase cleaves collagens to peptides that are unlikely to be identified by data bank searching when using the cleavage specificity of trypsin only. Despite these shortcomings, the direct analysis by LC-MS/MS of the human retinal vascular BMs is fast, economical and comprehensive, and yields certainly more information than any of the previous trial-and-error-based analyses using immunocytochemistry [40, 41].

### 2. Diabetes-specific differences in the proteomes of diabetic and non-diabetic vascular BMs

BMs from diabetic donors appear morphologically different from those from non-diabetic donors. Most striking, vascular BMs from diabetic donors are thicker than normal as shown by numerous publications [16–21]. This was also true for the samples that we used in our study, as shown in a previous publication using the same samples that were analyzed for this proteome analysis [21]. The morphological differences may suggest that the protein composition of diabetic and non-diabetic BMs is also different and let to this proteome analysis. Results from our study showed that all ECM proteins that were found for non-diabetic vascular BMs were also detected in vascular BMs from diabetic donors (Table 3; Fig 2). Vascular BMs of diabetic patients, however, included a series of proteins that were undetectable with the current technique in BMs from non-diabetic donors, such as two members of the complement family, C4 and C9. The BM sample from a 54 year old diabetic patient (54Di, Table 1) even included almost the entire series of complement proteins, such as C1q A and B, C3, C4, C5, C7, C8, and C9. The presence of major contributors to the complement cascade, including members of the complement attack complex, suggests a complement-mediated inflammation of the diabetic retinal vasculature. Complement had been associated with increased vascular permeability, leukocyte extravasation, and the loss of pericytes [42–44]. It is conceivable that the increased deposition of complement in retinal vasculature may contribute to the loss of retinal pericytes, a hallmark of vascular changes in diabetic retinas [13]. Other diabetes-specific proteins were ApoE, Timp3, fibrillin, fibronectin, and PRELP. The overexpression of fibronectin in diabetic vascular BMs had already been reported previously [21, 40, 41, 45]. All proteins previously mentioned are consistent with the assumption of a chronic inflammatory process in the retinal vasculature during long-term diabetes [46, 47].

Several proteins that are expressed in both diabetic and non-diabetic vascular BMs showed differential abundance in these two conditions. More abundant proteins were norrin, vitronectin and members of the collagen families IV, VI and XVIII, respectively. Norrin is a growth factor, which importantly contributes to vascular development [48] and is known to bind to BM-specific ECM proteins [49]. A deficiency of norrin causes X-linked exudative vitreoretinopathy, a defective development of intraocular blood vessel resulting in retinal detachment and eventually causes blindness [50]. The up-regulation and ECM-deposition of such a protein that promotes vascular growth may contribute to the retinal vascular rearrangement in long-term diabetes. Previous studies have shown that over-expression of norrin in murine lens did not induce sprouting of retinal vessels into the vitreous, rather promoted vessel growth and endothelial proliferation around the lens capsule [51, 52]. We speculate that excessive norrin that is expressed by Mueller glia cells only [53] may promote remodeling of existing capillaries within the retina, but is probably not responsible for the invasion of vessels into the vitreous during proliferative diabetic retinopathy. We speculate that the invasion of retinal vessels into the vitreous is a response to angiogenic growth factors secreted by the lens and mediated by the ECM of the vitreous [54]. One of the obvious candidates would be VEGF that is chemo-attractive for vascular sprouting and is expressed by the human lens [55].

Data analysis also indicated a different overall protein stoichiometry for vascular BMs harvested from diabetic or non-diabetic donors. Consistent with earlier results, diabetic BMs expressed more collagens and less laminins and proteoglycans [56]. A major characteristic of collagens is their high tensile strength, thus BMs with more collagens are presumably biomechanically stiffer than normal. Further, the lower abundance of proteoglycans effects a decreased water-binding capacity and implies a lower hydration of the BMs. Lower hydration means greater compaction of proteins within the BM matrix and, consequently, a stiffer BM. Previous studies have already shown that BMs from diabetic patients are stiffer than normal [21]. Further, diabetes has been related to an increase in BM stiffness *in vitro* [57], and analysis of vascular tissues from diabetic patients has linked diabetic affections to an increase in ECM stiffness [58, 59]. Surprisingly, our AFM stiffness data on vascular retinal BMs showed just the opposite.

We validated the up-regulated proteins based on the proteome studies in vascular BM whole mounts by immunocytochemistry. Data showed most proteins to be localized to microvascular aneurisms a ballooning of vascular walls that is characteristic to the diabetic vasculature in the retina [60]. It is currently unclear, whether the higher abundance of these proteins emerges along with the ontogeny of the vascular ballooning, or whether proteins are deposited or trapped post aneurism formation. An exception was norrin with a globally higher abundance in vascular BMs of diabetic retinas. Interestingly, norrin was not detected within the microvascular aneurisms, indicating that the aneurisms are not the origin of new and excessive vessel growth. It is important to note that vascular aneurisms had been detected in non-diabetic patients as well [61, 62]. However, their occurrence is limited to human donors beyond seventy years of age, well beyond the age of the samples that were examined in the current study.

### 3. Biomechanical properties of retinal vascular BMs

AFM probing of mounted capillaries by AFM showed that the outer surface of the vascular BMs from non-diabetic donor retina had a stiffness of 5.1 kPa, while the BM surface from diabetic donors was mechanically weaker with values ranging around 1.5 kPa. This finding was unexpected, since stiffness measurements from other BMs, such as the lens capsule and inner limiting membrane, showed an increase in stiffness after long-term diabetes. The lower stiffness of diabetic vascular BMs is also unexpected based on the proteome data that showed higher than normal collagen and lower than normal proteoglycans content in diabetic BMs. A higher abundance of both protein classes would indicate an increase in BM stiffness. However, the weaker vessel-BMs are consistent with the fact that blood vessels in diabetic patients have a high incidence of local ballooning to form microvascular aneurisms. Since cells have a stiffness that is by at least by a factor 10 lower than that of BMs, the ballooning of the retinal capillaries is most likely due a weaker than normal BM wall. A previous AFM study [23] has shown that the epithelial sides of ocular BMs are stiffer than the stromal sides. So far, we have not been able to obtain measurements of the inner surface of the vascular BMs. We, therefore, do not know whether the lower stiffness of the stromal side of the diabetic vascular BMs also applies to the epithelial/endothelial side. Accordingly, it is not clear whether, in accordance to a softening of the stomal side from vascular BMs, also the epithelial/endothelial surface of these structures will soften upon diabetic affection. Previous studies have shown that the two sides of several human ocular BMs, the epithelial and the stromal sides, have different surface properties [23]. In all cases, the collagen IV-rich stromal side was softer than the laminin-enriched epithelial side. The present finding would indicate that stiffness of BMs is not necessarily dependent on a high concentration of collagen IV. We also know from previous publications comparing the proteomes and biomechanical properties of ILM, vascular BMs, and lens capsule that the relative abundance of collagen IV is not the most decisive factor for BM stiffness [5, 23]. Results showed that the lens capsule with the highest concentration of collagen IV has an even lower stiffness than the laminin-rich ILM with relative low collagen IV concentrations. Even the Descemet’s membrane, with little collagen IV, has a similar stiffness as the collagen IV dominated lens capsule (Moes and Halfter, unpublished). The current data do not dispute the importance of collagen IV in BM stability, but show that collagen IV is not the only and most important factor for the biomechanical strengths of BMs.

In summary, this report introduces a straightforward procedure to analyze the protein composition of BMs. The simplified method allows for a direct comparis on of the proteomes from diabetic and non-diabetic BMs. Our results show that diabetic and non-diabetic BMs differ in two ways: First, we identify an array of proteins that prominently occur in diabetic BMs. Further, the stoichiometry of proteins in diabetic BMs differs from the composition of BMs from non-diabetic patients. Most of the putatively diabetes-related proteins were localized to microvascular aneurisms. We propose that several of these protein changes are indicators for the pathology of the vascular supply in diabetic retinas. Finally, we found that the vascular BMs had a softer wall, a fact that might explain the occurrence of aneurisms that are often found in retinal microvessels from diabetic donors.

## Supporting information

S1 Table Protein composition of retinal vascular BMs from a 47-year old non-diabetic donor. Sheet A: List of all proteins detected. Sheet B: List of the ECM proteins only and their relative concentrations in % and pie-chart.
(XLSX)

S2 Table Protein composition of retinal vascular BMs from a 54-year old non-diabetic donor. Sheet A: List of all proteins detected. Sheet B: List of the ECM proteins only and their relative concentrations in % and pie-chart.
(XLSX)

S3 Table Protein composition of retinal vascular BMs from a 64-year old non-diabetic donor. Sheet A: List of all proteins detected. Sheet B: List of the ECM proteins only and their relative concentrations in % and pie-chart.
(XLSX)

S4 Table Protein composition of retinal vascular BMs from a 38-year old diabetic donor. Sheet A: List of all proteins detected. Sheet B: List of the ECM proteins only and their relative concentrations in % and pie-chart.
(XLSX)

S5 Table Protein composition of retinal vascular BMs from a 52-year old diabetic donor. Sheet A: List of all proteins detected. Sheet B: List of the ECM proteins only and their relative concentrations in % and pie-chart.
(XLSX)

S6 Table Protein composition of retinal vascular BMs from a 54-year old diabetic donor. Sheet A: List of all proteins detected. Sheet B: List of the ECM proteins only and their relative concentrations in % and pie-chart.
(XLSX)

## Abbreviations

AFM: Atomic Force Microscopy
BM: basement membrane
ECM: extracellular matrix
LC/MS: liquid chromatography/mass spectrometry
LN: laminin
