# Correction: Diabetes-related changes in the protein composition and the biomechanical properties of human retinal vascular basement membranes

**DOI:** 10.1371/journal.pone.0195772

**Published:** 2018-04-06

**Authors:** Willi Halfter, Suzette Moes, Daphne O. Asgeirsson, Kathrin Halfter, Philipp Oertle, Esther Melo Herraiz, Marija Plodinec, Paul Jenoe, Paul Bernhard Henrich

There is an error in the sixth sentence of the first paragraph of the “Analysis of human retinal vascular BMs from non-diabetic donors” heading under the Results section. The correct sentence is: Results showed that the peptide yield from the three pairs of non-diabetic eyes ranged between 15 and 47μg total peptide (mean 29±16, n = 3; Table 1).

There are errors in the first and second sentences of the second paragraph of the “Analysis of human retinal vascular BMs from non-diabetic donors” heading of the Results section. The correct sentences are: Using a stringent 1% peptide false discovery rate cutoff, we identified between 45 and 110 proteins for these samples (mean 81±33, n = 3; S1–S3 Tables; each sheets A). Between 28 and 47 of the identified proteins were ECM constituents (mean: 36±10), accounting for 36 to 62% of the identified proteins (mean: 47±13%; Table 2 and S1–S3 Tables; each sheets B).

There is an error in the last sentence of the third paragraph of the “Analysis of human retinal vascular BMs from non-diabetic donors” heading of the Results section. The correct sentence is: The main non-ECM proteins were cytoskeletal constituents (15±10%), followed by nuclear (2.5±2.4%) and cytoplasmic proteins (6.9±1.1%; Table 2).

There is an error in the first sentence of the second paragraph of the “Distribution of diabetes-specific ECM proteins” heading of the Results section. The correct sentence is: A monoclonal antibody to the 7S domain of collagen IV α3 labeled the vascular BMs from both diabetic and non-diabetic eyes; staining of the microvascular aneurisms in the diabetic samples was weak.

The following information is missing from the Acknowledgements section: Work of MP and PO was supported by the Swiss National Science Foundation Nanotera Project awarded to the PATLiSci II Consortium.

The following information is missing from the Competing Interests statement: The University of Basel has filed patents (Method and device for controlling a scanning probe microscope: WO2014090971A1) related to the AFM technology based on the inventions of MP PO and RYHL.

Fig 5 is incorrect. The authors have provided a corrected version here.

**Fig 5:**
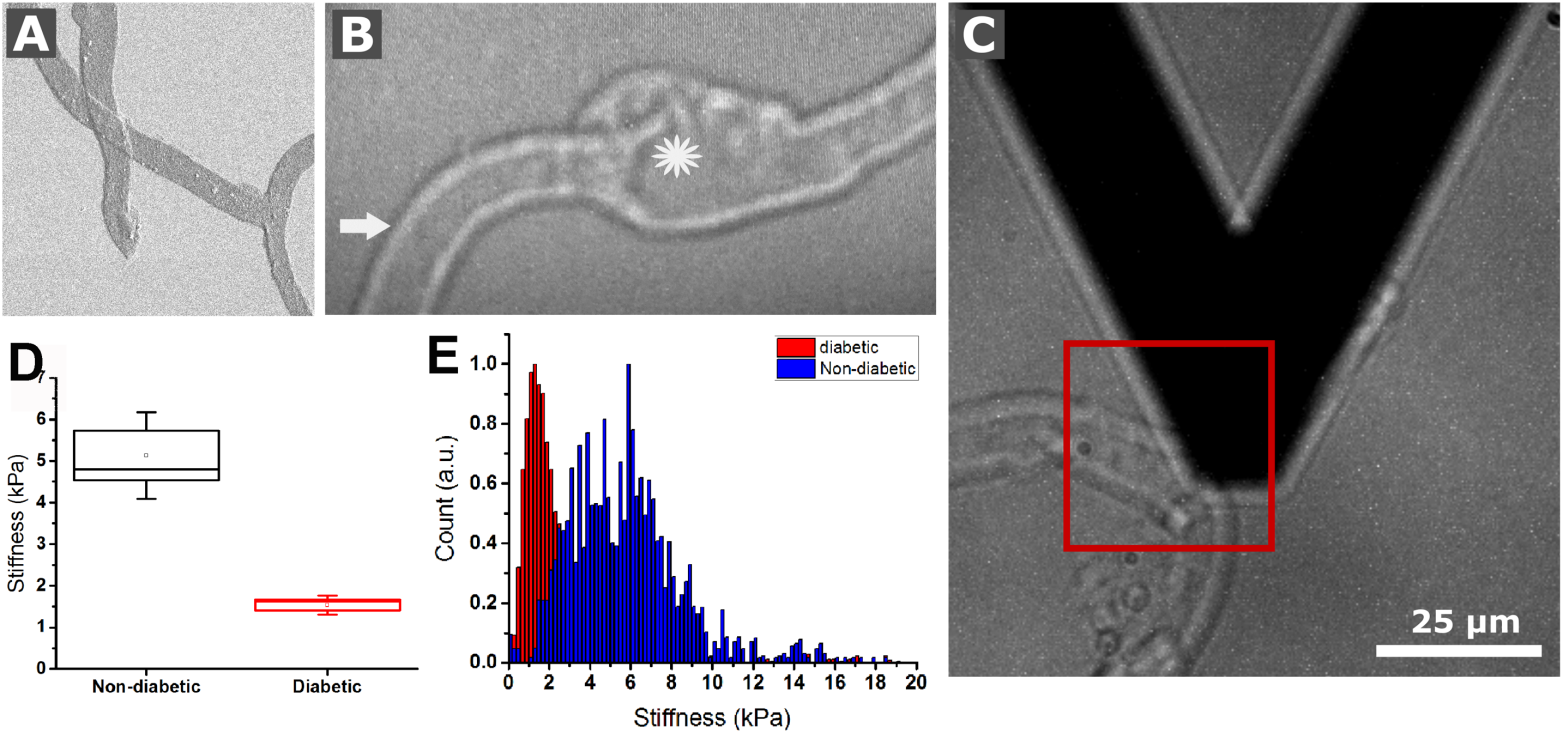
Biomechanical testing of human retinal vascular BM whole mounts by AFM. (A) Image shows a SEM micrograph of flat-mounted vascular BM sheets. (B) A representative light micrograph from a flat-mounted capillary during AFM measurements is shown. The star hereby marks an aneurism while the arrow indicates the region of AFM data collection on the sample surface in order to probe the biomechanical stiffness of a capillary. (C) Panel shows the AFM probe next to a capillary that was about to be probed in the indicated box. The box plot in (D) contrasts the average stiffness of non-diabetic and diabetic vascular BMs, respectively. Representative graphs show the relative frequency of AFM stiffness values for non-diabetic vascular BMs and diabetic vascular BMs (E).
